# Correlation between assisted reproductive technology-induced pregnancy and fetal cardiac anomalies

**DOI:** 10.5935/1518-0557.20210088

**Published:** 2022

**Authors:** Mahvash Zargar, Maryam Rahimi, Mojgan Barati, Farideh Moramazi, Abdolrahman Emami Moghadam, Parastoo Moradi Choghakabodi

**Affiliations:** 1 Department of Obstetrics and Gynecology; Fertility, Infertility and Perinatology Research Center, Ahvaz Jundishapur University of Medical Sciences, Ahvaz, Iran; 2 Fertility, Infertility and Perinatology Research Center, Ahvaz Jundishapur University of Medical Sciences, Ahvaz, Iran; 3 Department of Pediatric Cardiology, Ahvaz Jundishapur University of Medical Sciences, Ahvaz, Iran; Fertility, Infertility and Perinatology Research Center; 4 Ahvaz Jundishapur University of Medical Sciences, Ahvaz, Iran; Thalassemia and Hemoglobinopathy Research Center, Health Research Institute

**Keywords:** Assisted reproductive technology, heart defect, fetus

## Abstract

**Objective:**

To investigate the incidence of fetal heart defects in assisted reproductive technology (ART)-induced pregnancies compared to natural pregnancies as well as to detect their fetal and maternal risk factors associated with ART.

**Methods:**

In this retrospective cohort study, we collected data from the medical records belonging to 2877 pregnant women's fetuses, who underwent fetal echocardiography for various reasons, including ART, over the last 3 years.

**Results:**

There were no major cardiac anomaly in the ART-induced pregnancies, while it was seen in 1.32% of natural pregnancies; so, ART did not increase the risk of major cardiac anomalies. However, the incidence of fetal mild cardiac anomalies among fetuses derived from ART-induced pregnancies (51.43%) was significantly higher than that of natural pregnancies (44.43%, *p*=0.03). None of the ART-induced pregnancies had a history of a child with cardiac disease (vs. 7.56% in natural pregnancies). Also, the increased nuchal thickness (NT) and extra-cardiac anomalies were significantly more prevalent among natural pregnancies, indicating a significant negative correlation between ART and these two risk factors [χ2=10.24, r: -0.06, 95% CI(-0.0974 to -0.0221) and χ2=47.25, r: -0.129, 95% CI(-0.1656 to -0.0913), *p*<0.01, respectively]. The adjusted odds ratio of developing fetal mild cardiac anomalies were 1.37 times higher greater for ART-induced pregnancies compared to natural pregnancies [95% CI(1.072-1.769), *p*=0.01].

**Conclusions:**

Although the likelihood of developing fetal mild cardiac anomalies was 1.37 times higher for ART-induced pregnancy compared to natural pregnancy, ART did not increase the risk of major cardiac anomalies Although the likelihood of developing fetal mild cardiac anomalies was 1.37 times higher for ART-induced pregnancy compared to natural pregnancy, ART did not increase the risk of major cardiac anomalies.

## INTRODUCTION

The global prevalence of infertility increased by 0.370% and 0.291% per year for females and males from 1990 to 2017, respectively. This increasing global burden of infertility veered towards assisted reproductive technology (ART) development and registries (Sun *et al*., 2019). Well-known assisted reproductive technologies are in vitro fertilization (IVF), intra-cytoplasmic sperm injection (ICSI), GIFT, and zygote intra-fallopian transfer (ZIFT) (Giorgione *et al*., 2018).

ART is usually safe, and most pregnancies conceived by ART lead to favorable outcomes. However, there are some reports on the increased risk of birth defects in infants conceived by ART (Hansen *et al*., 2013; Wen *et al*., 2012; Pandey *et al*., 2012). Other studies reported the same risks for birth defects related to embryo quality in ART. A higher risk of birth defects has been found among fetuses conceived through fresh embryo cycles, compared with frozen embryo-related births and spontaneously conceived births (Davies *et al*., 2012). The blastogenesis defects were also reported to be three times higher in ART births following fresh embryo transfer (but not frozen-thawed embryo transfers) *vs*. non-ART births (Halliday *et al*., 2010). However, there is no sufficient valid evidence on the risk of birth defects associated with other ART procedures, such as donor oocytes (Gupta *et al*., 2014) and assisted hatching (Jwa *et al*., 2015).

Congenital heart defects (CHDs), as the most common birth defect, is the main cause of perinatal mortality, being present in 6 per 1000 live births (Hoffman & Kaplan, 2002). Based on a review of twenty studies on ART-related CHD since 1980, the incidence of mild CHD in ART pregnancies (2.2%) was higher than that in non‐ART pregnancies (1%). The incidence of severe CHD in naturally conceived pregnancies and ART pregnancies was 1.2% and 1.4%, respectively (Patil *et al*., 2018). On the contrary, other studies report no association between ART (IVF/ICSI) and CHD, even after adjusting for maternal age and multiple gestations (Schofield *et al*., 2017; Votava-Smith *et al*., 2014). The echocardiography practice guidelines have not yet reached a consensus on whether ART conception should be considered as an indication for fetal echocardiography or not (Giorgione *et al*., 2018). A definitive answer deserves further investigations on this area. So, our study has investigated the incidence of fetal cardiac anomalies in ART-induced pregnancies as well as in natural pregnancies.

## MATERIAL AND METHODS

### Study design and data collection

This retrospective cohort study (from March 2016 to March 2019) comparatively evaluated the incidence of fetal CHD and its various types in women with natural pregnancy and ART-induced pregnancy. The inclusion criteria involved the fetuses submitted to fetal echocardiography for different causes in the last three years. We assessed a series of fetal and maternal risk factors for fetal echocardiography, including cardiac and non-cardiac abnormalities, maternal age older than 35 years, fetal nuchal thickness (NT) above the 95^th^ percentile, having a child with a history of CHD, maternal rheumatism and/or diabetes. Patients with vague echocardiography images due to various reasons, e.g., a history of severe obesity or abdominal surgery, and patients with incomplete information were not included.

The data from the fetuses and their mothers were collected from their medical records, including demographic data, gestational age, abnormal fetal NT, history of CHD, maternal underlying diseases, male or female infertility cause, and abnormal cardiac and non-cardiac findings.

### Sample size

We used the census method to prepare our sample, i.e., all eligible patients with adequate documents who underwent fetal echocardiography in the Perinatology department in the last 3 years were studied.

### Statistical analysis

The qualitative variables were expressed as frequency (percentage), while the quantitative or descriptive variables were described as mean, standard deviation, median and interquartile range. We checked the data normality by the Shapiro-Wilk test, and the relationship between qualitative variables and fetal cardiac anomalies was examined by the Chi-square test. The odds ratios (ORs) were used to estimate relative risk in the logistic regression model, and all risk factors were included in the model too. A *p*<0.05 is considered statistically significant and the data was analyzed by the SPSS version 26.

### Ethical considerations

All the procedures in this study involving human participants were in accordance with the ethical standards of the national research committee and the 2008 Helsinki declaration, and its later amendments or comparable ethical standards [Ethical Code: IR.AJUMS.REC.1398.682]. All patient information is confidential.

## RESULTS

### Baseline Characteristics

Table 1 shows the demographic and clinical data. From a total of 2,857 fetuses, 280 cases have arisen from ART-induced pregnancies *vs*. 2,577 natural pregnancies. Mothers who conceived by ART were usually older than those who became naturally pregnant (*p*<0.001). Also, mothers who underwent ART had a higher body mass index (BMI) (*p*<0.001).

**Table 1.** Demographic information and comparison of the frequency of the pregnancy-related variables between two groups, i.e., Natural pregnancy and ART-induced pregnancy.

Variables	All cases (n=2857)						
	Natural pregnancy [n=2577 (90.2%)]	ART-induced pregnancy [n=280 (9.8%)]	*p*	Classification of ART cases based on the causes of infertility			
				Male factor	Female factor	Other	Both
**AGE [n(%)]** < 25 25≥ ≤35 > 35	324 (12.57) 1625 (63.06) 628 (24.37)	14 (5) 162 (57.86) 104 (37.14)	**<0.001_a_**	7 (2.50) 63 (22.50) 28 (10)	1 (0.36) 30 (10.72) 24 (8.57)	6 (2.14) 60 (21.43) 42 (15)	0 9 (3.21) 10 (3.57)
**BMI [n(%)]** < 18 18-24.9 25-29.9 30-40	Missed data: 26 72 (2.80) 1185 (45.98) 771 (29.92) 523 (20.30)	Missed data: 6 6 (2.14) 107 (38.21) 106 (37.86) 55 (19.64)	<0.001	3 (1.07) 36 (12.86) 38 (13.57) 18 (6.43)	1 (0.36) 21 (7.50) 22 (7.86) 10 (3.57)	1 (0.36) 43 (15.36) 39 (13.93) 24 (8.57)	1 (0.36) 7 (2.50) 7 (2.50) 3 (1.07)
**Have child with cardiac disease [n(%)]** No Yes	2382 (92.43) 195 (7.57)	280 (100) 0	<0.001	98 (35) 0	55 (19.64) 0	108 (38.57) 0	19 (6.79) 0
**Increased NT [n(%)]** No Yes	2365 (91.77) 212 (8.23)	272 (97.14) 8 (2.86)	<0.001	96 (34.29) 2 (0.71)	54 (19.29) 1 (0.36)	104 (37.14) 4 (1.42)	18 (6.43) 1 (0.36)
**Extra-cardiac anomalies in ultrasound [n(%)]** No Yes	2022 (78.46) 555 (21.54)	268 (95.71) 12 (4.29)	<0.001	94 (33.57) 4 (1.43)	52 (18.57) 3 (1.07)	103 (36.78) 5 (1.79)	19 (6.79) 0
**Type of extra-cardiac anomalies:** No obvious non-cardiac anomalies CPC SUA Cardiac echogenic focus Pyelectasis Echogenic Bowel CPC + SUA Cardiac echogenic focus +CPC Pyelectasis +CPC Cardiac echogenic focus +SUA Echogenic Bowel + SUA Cardiac echogenic focus + Pyelectasis Cardiac echogenic focus+ Pyelectasis + echogenic bowel	2021 (78.42) 234 (9.08) 58 (2.25) 177 (6.87) 45 (1.75) 6 (0.23) 2 (0.08) 11 (0.43) 14 (0.54) 2 (0.08) 1 (0.04) 5 (0.19) 1 (0.04)	268 (95.71) 3 (1.07) 1 (0.36) 6 (2.14) 1 (0.36) 0 0 0 1 (0.36) 0 0 0 0	<0.001 0.06 <0.01 0.13 ... ... ... ... ... ... ... .... ...	94 (33.57) 0 1 (0.36) 1 (0.36) 1 (0.36) 0 0 0 1 (0.36) 0 0 0 0	52 (18.57) 0 0 3 (1.07) 0 0 0 0 0 0 0 0 0	103 (36.79) 3 (1.07) 0 2 (0.71) 0 0 0 0 0 0 0 0 0	19 (6.78) 0 0 0 0 0 0 0 0 0 0 0 0
**Mother’s diabetes** No Yes	2272 (88.16) 305 (11.84)	253 (90.36) 27 (9.64)	0.32	88 (31.43) 10 (3.57)	48 (17.14) 7 (2.50)	98 (35) 10 (3.57)	19 (6.79) 0
**Mother’s rheumatic diseases** No Yes	2540 (98.56) 37 (1.44)	279 (99.64) 1 (0.36)	0.22	97 (34.64) 1 (0.36)	55 (19.64) 0	108 (38.57) 0	19 (6.79) 0
**Major cardiac anomaly** No Yes Mild cardiac anomaly	1432 (55.57) 34 (1.32) 1111 (43.11)	136 (48.57) 0 144 (51.43)	0.08 0.009	57 (20.36) 0 41 (14.64)	23 (8.21) 0 32 (11.43)	48 (17.14) 0 60 (21.43)	8 (2.86) 0 11 (3.93)
**Fetal Non-major Cardiac Anomalies** no VSD ASD Arrhythmia VSD + ASD VSD + Arrhythmia VSD + ASD	1503 (58.32) 1045 (40.55) 3 (0.12) 20 (0.78) 1 (0.04) 5 (0.19) 0	137 (48.93) 140 (50) 0 0 0 2 (0.71) 1 (0.36)	0.003 0.67 0.27 0.17 0.30 0.17	57 (20.35) 40 (14.29) 0 0 0 1 (0.39) 0	24 (8.57) 31 (11.07) 0 0 0 0 0	48 (17.14) 58 (20.71) 0 0 0 1 (0.36) 1 (0.36)	8 (2.86) 11 (3.93) 0 0 0 0 0
**Fetal echocardiographs** Medical indication by parents	1987 (77.10) 590 (22.90)	195 (69.64) 85 (30.36)	0.006	63 (22.50) 35 (12.50)	41 (14.64) 14 (5)	76 (27.14) 32 (11.43)	15 (5.36) 4 (1.43)

a: Comparison of pregnancy frequency in different age ranges

ART= Assisted reproductive technology.

BMI= Body mass index

NT= Nuchal thickness

CPC= Choroid plexus cysts

SUA= Single umbilical cord artery

VSD: Ventricular septal defect

ASD: Atrial septal defect

Most cases in the study had no children with cardiovascular disease (92.52%), and no cardiac and extra-cardiac anomalies seen in the ultrasound (54.50% and 79.60%, respectively). Also, most cases had normal NT (91.65%) and were non-diabetics (87.76%) and did not have rheumatoid disease (97.98%). Most fetal echocardiographs were done by medical indications (75.84%) and the rest by parent request (23.46%). There was no significant correlation between maternal basic disease (rheumatic diseases and diabetes) and ART (*p*>0.05).

### Association of ART with maternal and fetal risk factors

About 7.56% of naturally pregnant women had at least one child with cardiac disease, while none of the ART-induced pregnant women had a child with cardiac disease. The male-related factor was the most prevalent cause of infertility among the women who underwent ART. The increased nuchal thickness was significantly more prevalent among natural pregnancies [8.22% *vs*. 2.85% in ART-induced pregnancies; *p*<0.001]. Also, there was no correlation between the increased NT and major fetal cardiac anomalies (*p*=0.80). A significant negative correlation was found between increased NT and ART-induced pregnancies [χ^2^=10.24, *p*=0.001; r: -0.06, 95% CI (-0.0974 to -0.0221)].

Extra-cardiac anomalies, especially choroid plexus cysts (CPC), were more prevalent in natural pregnancies [21.54% *vs*. 4.29% in ART-induced pregnancies; Table 1], and there was a negative correlation between ART and extra-cardiac anomalies [χ^2^=47.25, *p*<0.001; r: -0.129, 95% CI (-0.1656 to -0.0913)].

### Association of ART with Fetal Cardiac Anomalies

The incidence rate of fetal mild cardiac anomalies among fetuses derived from ART-induced pregnancies (51.43%) was significantly higher than that of natural pregnancies [44.43%, *p*=0.03; Table 2]. No major cardiac anomaly was found in ART-induced pregnancies while it was seen in 34 cases (1.32%) of the natural pregnancy group; however, this difference was not statistically significant (*p*=0.08). Ventricular septal defect (VSD) was the most prevalent type of mild cardiac anomaly in both studied groups, with a significantly higher incidence rate in ART-induced pregnancies [50% *vs*. 40.55%, *p*=0.003]. All fetuses suffering from major cardiac anomalies were from the natural pregnancy group; therefore, ART did not increase the risk of major cardiac anomalies.

**Table 2.** Unadjusted and Adjusted Odds Ratios and 95% CIs for Fetal Mild Cardiac Anomalies based on Logistic Regression.

	Unadjusted OR (95% CI)	*p*-value	Adjusted OR (95% CI)	*p*-value*
**ART**	1.397 (1.091-1.789)	0.008	1.377 (1.072-1.769)	0.01
**Age > 35 years old**	1.097 (0.927-1.299)	0.28	_	_
**BMI > 25**	0.994 (0.856-1.153)	0.93	_	_
**History of child with cardiac disease**	1.373 (1.027-1.837)	0.03	1.370 (1.020-1.838)	0.03
**Increased NT**	1.181 (0.896-1.555)	0.23	_	_
**Mother’s diabetes**	0.988 (0.785-1.245)	0.92	_	_
**Mother’s rheumatic diseases**	0.585 (0.294-1.164)	0.127	0.795 (0.656-0.962)	0.01
**Extra-cardiac anomalies**	0.755 (0.625-0.911)	0.003		

NT= Nuchal thickness

* *p*-value from multivariable adjusted logistic regression

Based on the results of univariate logistic regression, age >35 years old, BMI >25, maternal baseline diseases, and increased NT were not confounders of the association between ART and mild fetal cardiac anomalies (Table 2). Although a "history of a child with cardiac disease" and "Extra-cardiac anomalies" is suspected as negative confounders based on the univariate logistic regression, they did not show any significant negative effects on the association between ART and mild fetal cardiac anomalies based on the results of multivariate analysis. The results of the univariate logistic regression showed a strong association between ART and mild fetal cardiac anomalies [unadjusted OR: 1.397 (1.091-1.789), *p*=0.008], and according to the results of the second analysis, this strong association was still evident, despite the slight negative impact of the risk factors [Adjusted OR: 1.377 (1.072-1.769), *p*=0.01; Table 2].

## DISCUSSION

This study found that fetuses derived from ART-induced pregnancies were not at higher risk of having major cardiac anomalies compared to those who were derived from a natural pregnancy. However, there was a strong association between ART and mild fetal mild cardiac anomalies, regardless of the slight negative effects of "Extra-cardiac anomalies" and "history of a child with cardiac disease". The history of a child with a cardiac disorder may increase the risk of fetal cardiac anomalies in the next pregnancies. Our findings are somewhat confirming the findings of Wen *et al*. (2020). They found a remarkable association between ART pregnancy and a high risk of CHD. However, this strong association decreased after adjusting for several risk factors simultaneously (adjusted OR, 1.70; 95% CI: 1.48-1.95). In fact, the real direct association between ART pregnancy and CHD in singleton pregnancies was 1.09 (95% CI, 0.93-1.25), and 87.3% of this strong association was mediated by twinning. By contrast, our findings didn't find any significant association between ART and major fetal cardiac anomalies; yet ART was strongly associated with mild cardiac anomalies. Nevertheless, the detection of major cardiac anomalies is more vital than mild cardiac anomalies because the mild anomalies are mostly curable and or have no hemodynamic impact.

Based on a retrospective cohort study (2006-2016) ran by Yang *et al*. (2018) on 112,043 pregnant women and 114,522 newborns, ART-conceived infants had a higher probability of having any of the birth defects (adjusted OR: 2.10 (95% CI: 1.63-2.69). Also, ART-conceived infants are more prone to developing musculoskeletal, gastrointestinal, urogenital, respiratory, and cardiovascular defects. Yang *et al*. (2018) and Wen *et al*. (2020), proved the mediating effect of multiple pregnancies on ART-related birth defects. However, the findings of Yang *et al*. (2018) have also shown that the sum of individual effects of ART and twins on birth defects were higher than their combined effects. Nevertheless, some studies reported that the high risk of birth defects following ART is attributed to maternal infertility issues (Huang *et al*., 2015; Yang *et al*., 2014; Moore Simas *et al.,* 2019). Our findings did not show any significant association between maternal baseline diseases (i.e., diabetes and rheumatoid disease) and fetal major cardiac anomaly, which was not consistent with a lot of preceding reports (Lisowski *et al*., 2010; Jaeggi *et al*., 2001).

We investigated various types of fetal cardiac and extra-cardiac anomalies among fetuses derived from ART and non-ART pregnancies, respectively. Based on our findings, the adjusted Odds ratio of developing mild fetal cardiac anomalies were 1.37 times higher for ART-induced pregnancy compared to natural pregnancies. Based on Iwashima *et al*. (2017), there was no significant difference between the spontaneous conception group and assisted conception group in terms of severe CHD (*p*=0.892); and ART did not increase the risk of CHD; our findings are consistent with those from Iwashima *et al*. (2017). In our study, mild cardiac anomalies, particularly VSD, were responsible for 100% of the association between ART-induced pregnancy and fetal cardiac anomalies. Extra-cardiac anomalies, particularly CPC, were more prevalent among fetuses derived from natural pregnancies, and the risk of developing fetal extra-cardiac anomalies seems to be less in ART-induced pregnancy compared to natural pregnancy. It may be because of the timely use of pre-gestational genetic diagnosis (PGD) methods during ART, which discards fetuses with chromosomal congenital heart diseases, and serious genetic disorders, especially numerical chromosomal abnormalities such as Down and Turner syndromes.

Also, our findings showed that the increased NT was remarkably more prevalent in natural pregnancies. There was no association between the increased NT and major fetal cardiac anomalies, and most fetuses derived from ART-induced pregnancies had normal NT. In this regard, our results were in contrast with the results from Hui *et al*. (2005) and Riestenberg *et al*. (2021); Hui *et al*. (2005) retrospective study on 16,673 spontaneous pregnancies indicated a significant increase in median NT in fetuses conceived by IVF and/or ICSI pregnancies. So, they concluded that Increased NT in ART-dependent pregnancies may be due to a delay in fetal growth and/or adverse antenatal course. In this regard, Riestenberg *et al*. (2021) did not find any significant difference in the rate of abnormal NT and fetal anomalies between IVF pregnancies and natural conceptions. However, the rates of abnormal second trimester serum analytes and placental ultrasound abnormalities were significantly more evident among patients conceived by IVF, compared to those conceived naturally (Riestenberg *et al*., 2021). Based on our results, although mothers who were naturally pregnant had better physical health status, the rate of the increased NT and extra-cardiac anomalies were remarkably higher among them compared to ART-induced pregnancies. Contradictory findings in various studies may imply the different effects of non-studied risk factors in different geographical areas on pregnancy outcomes. For example, in our studied the city population is more exposed to air and water pollution, which has harmful impacts on general health and fertility, as per previously reported (Rashidi *et al*., 2015).

## CONCLUSION

Although the history of cardiac disorders in the previous children and extra-cardiac anomalies were associated with an increased incidence of major fetal cardiac anomalies, ART had a negative correlation with these risk factors, and it did not increase the risk of major cardiac anomalies. Although all fetuses suffering from major cardiac anomalies were from the spontaneous pregnancy group, the likelihood of developing mild fetal cardiac anomalies was 1.37 times higher for ART-induced pregnancy compared to natural pregnancy. Maternal age, maternal BMI, abnormal fetal NT, maternal diabetes, and rheumatic disease had no association with major fetal heart anomalies. The favorable consequences of ART in this study may be due to the use of the pre-gestational genetic diagnosis (PGD) methods, which can discard fetuses affected by serious genetic and heart disorders. In this regard, we recommend more studies for evaluating other ART-related risk factors, e.g., a multicenter study on the impact of air pollution on ART outcomes.

## Abbreviations

ART: assisted reproductive technology
GIFT: gamete intra-fallopian transfer
IVF: in vitro fertilization
ICSI: intra-cytoplasmic sperm injection
ZIFT: zygote intra-fallopian transfer
CHD: congenital heart defects
NT: nuchal thickness
BMI: body mass index
SUA: single umbilical cord artery
VSD: ventricular septal defect
CPC: choroid plexus cysts
PGD: pre-gestational genetic diagnosis
